# The rapid CD4 + T-lymphocyte decline and human immunodeficiency virus progression in females compared to males

**DOI:** 10.1038/s41598-020-73852-0

**Published:** 2020-10-08

**Authors:** Nader Parsa, Pari Mahlagha Zaheri, Ross G. Hewitt, Ali Karimi Akhormeh, Samira Taravatmanesh, Lisa Wallin

**Affiliations:** 1 Cardiovascular Research Center, Shiraz University of Medical Sciences, Shiraz, Iran; 2 Shiraz University of Medical Sciences, Shiraz, Iran; 3 University at Buffalo, State University of New York, Buffalo, NY USA; 4 Strong Memorial Hospital, University of Rochester, Rochester, NY USA

**Keywords:** HIV infections, Prognostic markers, Epidemiology, Infection

## Abstract

CD4 + T-lymphocyte counts are used to assess CD4 + decline and the stage of human immunodeficiency virus (HIV) progression in HIV-infected patients. Clinical observation suggests that HIV progress more rapid in females than males. Of the original 5000 HIV-infected population of Western New York HIV/AIDS, Referral Center at Erie County Medical Center (ECMC), 1422 participated in the cohort study. We identified 333 HIV-infected patients with CD4 + T-cell-counts ≥ 500/µƖ, among them 178 met the inclusion criteria for the 10-year study. Females had higher mode (600 vs. 540) and mean (741.9 vs. 712.2) CD4 + counts than males at baseline. However, CD4 + declined faster among females in a shorter time than males (234.5 vs. 158.6, *P* < 0.004), with rapid HIV progression. Univariate analyses determined that females had a 40% higher risk for CD4 + decline than males. The bivariate analyses specified CD4 + decline remained greater in females than males. Multivariate analyses which employed Cox’s proportional Hazard-Model to adjust for numerous variables simultaneously identified women had almost twice the risk for CD4 + decline and rapid HIV progression than males (RR = 1.93; 95%CI 1.24, 2.99). Although the biological mechanism remains unknown, findings suggest gender differences in CD4 + decline, with a higher risk of rapid HIV progression and shorter longevity in females.

## Introduction

HIV is a contagious, deadly disease that has rapidly spread worldwide^1^. It is a member of the lentivirus group of retroviruses that specifically infects T-lymphocyte white blood cells (WBC) with CD4 + surface receptors^2^. The HIV virus then attacks CD4 + T-cells, kills them, decreases immune function, and leads to opportunistic infections (**OI’s**) and rare cancers. It is very rare for OI’s to occur when the CD4 + T-lymphocyte count is ≥ 500µƖ^3^. Within a week or two of infection, p24 antigen appears in the blood, followed in about 6–10 weeks by p24 antibodies.

Normally replication of HIV virus is greater than several hundred million per day^4^. The strongest implication associated for HIV progression is the depletion of CD4 + T-lymphocytes in the immune system^3^. The half-life of the virus is two days, with approximately 30% of virus replication occurring in 24-h, and complete turnover of the entire virus population every 14 days^5^. The rate of CD4 + T-lymphocyte decline can vary but averages 40–80 cells/µƖ/year^6^.

The 2019 Centers for Disease Control (CDC) and Prevention reported females in 2016 were more than 20% of HIV-infected persons in the United States^7^. The World health organization (WHO) and the United Nations Program on AIDS (UNAIDS) identified nearly 50% (18.8 million) of HIV-infected patients worldwide are females^8^.

The significant gender differences such as gender-based beliefs, unfavorable cultural diversity, violence, lack of accessing care and so on, make women in particular more vulnerable to becoming HIV-infected in ways that correspond to their risk perception^9,10^. Due to socio-demographic and socio-behavioral status, females appeared to be more susceptible to increase risk of CD4 + decline and HIV progression compared with males^11^.

Previous studies identified that females had a higher CD4 + T-cell count^12–18^ at the beginning of infection compared to males. In other studies, gender differences showed rapid CD4 + decline and HIV progression in females compared to males^12,14,19–21^. Therefore, for any given CD4 + T-cell count, females may be at a higher risk of HIV progression and female gender by itself, appears to increase risk of HIV progression compared to males. Consequently, the aim of this study was to examine the possible gender difference in CD4 + T-lymphocyte decline and HIV progression.

## Material and methods

### Design

This is a longitudinal cohort study conducted on HIV-infected patients that excluded patients with the following criteria: 1) If they were placed on any type of combination antiretroviral medication or protease inhibitors (***PI’s***) that may interfere with the natural history of progression, 2) CD4 + T-lymphocyte count˂500/µƖ, 3) pregnant woman at entry or within the first 6 months of the study, 4) diagnosed with non-HIV-immunosuppression, 5) patients with signs/symptoms of **OI**’s, and 6) who die within the first 6 months of the study. However, inclusion criteria were HIV-infected subject with CD4 + T-lymphocyte counts ≥ 500/µƖ without above exclusion criteria.

### Study population

The target population was 5000 HIV/AIDS-infected subjects served through initial visits and continuing care revisits from diverse socioeconomic, ethnic, and racial groups, by the immunodeficiency department, who attended the WNY HIV/AIDS Referral Center at ECMC over a 10-year period.

### Ethical considerations

This study was in accordance with the Health Sciences Institutional Review Board (HSIRB) of the State University of New York, Buffalo Institutional Review Board (SUNYBIRB) Permissions and had been approved at the WNY HIV/AIDS Referral Center at ECMC, related to any unanticipated problems involving risk to human subject and any serious adverse events before data collection.

### Sample size

Of the original population 1,422 participants (1,111 males, 311females), 333 HIV-infected patients were identified with a CD4 + T-cell count ≥ 500/µƖ (231 males, 102 females). After deletion of patients who met specific exclusion criteria, 178 HIV-infected subjects with CD4 + T-lymphocyte counts ≥ 500/µƖ corresponding to CDC stage-***A1*** definition as followed remained for the 10-year study evaluation in Table 1^22^.

Most patients were diagnosed with HIV initially at the ECMC private practice clinics, the Women’s Clinic, and at the Immunodeficiency Department. Less than 5% of study patients who were referred from other facilities were followed by this department.

In this study gender as the independent variable was assessed by medical record review of cases during the study period. Study participants were divided by gender for analyses. The outcome variable was CD4 + T-lymphocyte decline as a precursor to HIV progression according to International Classification of Disease, **10th** revision **(ICD-10)**^23^.

The ECMC Immunodeficiency Department is a major referral center in WNY for HIV detection and treatment with well-trained staff and advanced clinical diagnostic laboratory were used for study. Diagnosis of eligible HIV-infected subjects was ascertained by sero-immunologic techniques, such as EIA^24,25^, ELISA and confirmed by Western Blot^25–27^, to detect HIV antibodies. The CD4 + T-cell counts were confirmed by flow-cytometry technique in the tertiary level (level-3) certified laboratory of ECMC and recorded in the medical file. Eligible HIV-patients were followed for at least 6 months within the study period. Pregnant patients were included in the study if the pregnancy occurred after the first 6 months of investigation.

### Covariates

Several covariates such as **a**) clinical; **b**) hematology (Complete blood cell count (CBC)/ Differential automated (*Diff*.); **c**) socio-demographic (supplemental Tables 1, 2) and; **d**) socio-behavioral risk factors (supplemental Tables 3, 4) were used to control for possible confounding or interaction.

**Table 1:** CDC stages definition.

CD4 + T-Cell	A1	B1	C1
CD4 + T-lymphocyte counts ≥ 500/µƖ	Asymptomatic HIV-infection, Acute primary HIV-infection with illness or history of acute HIV-infection or PGL*	Symptomatic conditions but not with acute HIV-infection, PGL or any HIV indicator conditions	With AIDS indicator conditions

*PGL persistent generalized lymphadenopathy.

**Table 2:** Test Results with Equal Variances for CD4 Base Value, Length of Follow-up, and CD4 Decline during the Follow-up Time for HIV-Infected Males (n = 118) Compared with HIV-Infected Females (n = 60).

Variable	Gender		Statistical analysis				
	Male (n = 118)	Female (n = 60)					
	Mean ± SD	Mean ± SD	Mean Diff	SE Diff	95%confidence interval	*t*-test	Sig (2-tailed)
CD4 base value (µƖ)	712.2 ± 186.6	741.9 ± 301.5	− 29.6	36.72	(− 102.11, 42.84)	− 0.807	0.421
Length of time (months) from initial HIV + diagnosis to First CD4 count	7.20 ± 16.31	7.45 ± 15.17	− 0.2805	2.528	(− 5.27, 4.71)	− 0.1	0.912
Follow-up length (months)	25.5 ± 17.4	21.9 ± 16.9	3.58	2.74	(− 1.83, 8.98 )	1.318	0.193
CD4 decline (µƖ) during the follow-up time	158.6 ± 120.3	234.5 ± 204.6	− 75.9	26.32	(− 127.93,− 23.93)	− 2.89	0.004

Diff, Difference; Sig, Significance.

**Table 3:** Compared non-rapid progressor and rapid progressor of CD4 decline status during the follow-up time for HIV-infected males and females.

Variable		Male % (n = 118)	Female % (n = 60)	Total % (n = 178)	χ^2^	*p *value
CD4 Decline Status:	Non-rapid progressor(CD4decline < 200/µƖ)	75.4 (89)	58.3 (35)	70.0 (124)	6.0	0.02
	Rapid Progressor (CD4 decline > 200 /µƖ)	24.6 (29)	41.7 (25)	30.0 (54)		
	Total	100.0 (118)	100.0 (60)	100.0 (178)

**Figure 1:**
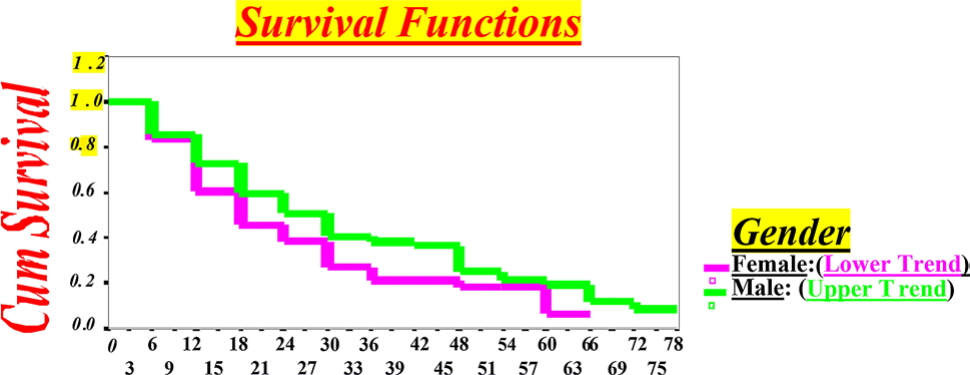
CD4 + Decline by Months for Western New York HIV-infected Subjects up to Endpoint (< 500/µL) in HIV-infected Females compared to Males by Survival Functions Model.

**Figure 2:**
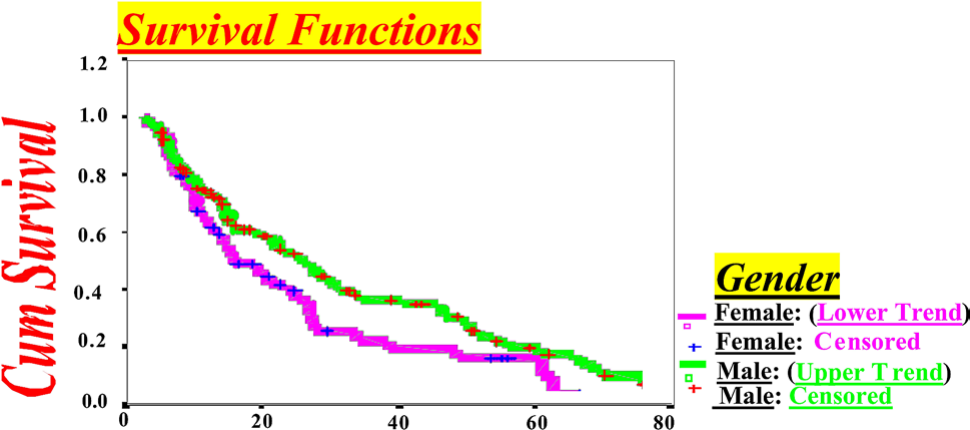
CD4 + Decline by Months for Western New York HIV-infected Subjects up to Endpoint (< 500/µL) in HIV-infected Females compared to Males by Survival Functions Model.

**Figure 3:**
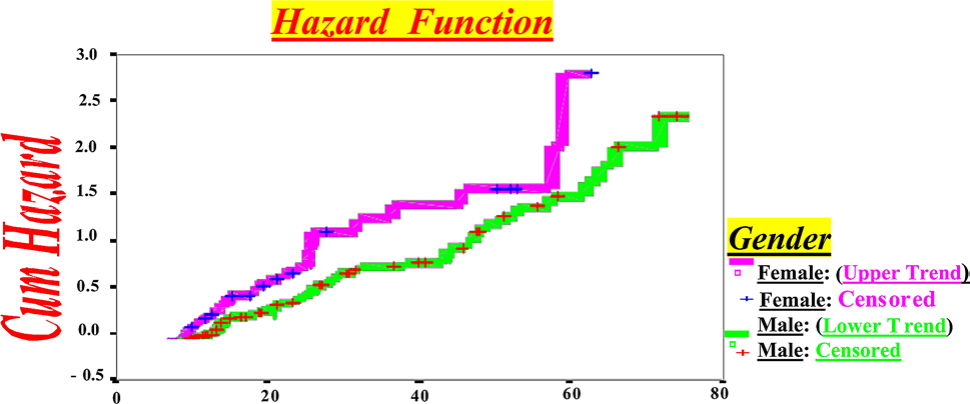
CD4 + Decline by Months for Western New York HIV-infected Subjects up to Endpoint (< 500/µL) in HIV-infected Females compared to Males by Hazard Function Model lower women's longevity.

**Table 4:** Test results with equal variances for laboratory-haematology: white blood cell (k/cumm), lymphocyte percent and platelet (k/cumm), results for HIV-infected males (n = 118) compared with HIV-infected females (n = 60).

Variable	Gender		Statistical analysis				
	Male(n = 118)	Female (n = 60)					
	Mean ± SD	Mean ± SD	Mean Diff	SE Diff	95% Confidence Interval	t-Test	Sig. (2-tailed)
White Blood Cell (WBC) k/cumm	6.7 ± 1.8	6.1 ± 1.8	0.541	0.285	(− 0.02, 1.10)	1.899	0.059
Lymphocyte Percent	36.9 ± 11.9	35.9 ± 12.2	0.999	1.895	(− 2.74, 4.74)	0.527	0.599
Platelet k/cumm	251.0 ± 69.0	280.0 ± 75.0	− 29.33	11.142	(− 51.32, − 7.34)	− 2.63	0.001

Diff., Difference; Sig., Significant.

**Table 5:** Univariate, bivariate results of female gender and started CD4 + T-Cell base value modeling and multivariate modeling and CD4 + T-Cell Base value simultaneously with other possible important covariates associated with CD4 + decline toward to HIV progression during study period.

Analysis	Models	Variable	Statistical Analysis								
			n	Events % (n)	Censored % (n)	β(Beta)	SE(β)	[β/SE(β)]^2^	Sig. (P-Value)	HR (eβ)	95% CI for eβ
Univariate	Model-1	Gender:	178	67 (119)	33 (59)						
		1. Males	118			0	1	Referent	0	1	Referent
		2. Females	60			0.343	0.194	3.114	0.0700	1.4	0.96, 2.06
	Model-2	CD4 + T-Cell Base Value (in Hundreds)	178	67 (119)	33 (59)	− 0.255	0.066	14.674	0.0001	0.9	0.68, 0.88
Bivariate	Model-1	Gender:	178	67 (119)	33 (59)						
		1. Males	118			0	1	Referent	0	1	Referent
		2. Females	60			0.352	0.194	3.279	0.0701	1.4	0.97, 2.08
		CD4 + T-cell base value (in hundreds)	178	67 (119)	33 (59)	− 0.251	0.066	14.319	0.0002	0.8	0.68,0.88
Multivariate	Model-1	Gender:	178	69 (119)	33 (59)						
		1. Males	118			0	1	Referent	0	1	Referent
		2. Females	60			0.657	0.223	8.628	0.0033	1.93	1.24, 2.99
		CD4 + T-Cell Base Value (in Hundreds)	178	67 (119)	33 (59)	− 0.387	0.093	17.313	0.0001	0.7	0.56, 0.81
		Gender & CD4 + T-Cell base value (in Hundreds) and other covariates* in study	178	67 (119)	33 (59)	0.281	0.117	5.779	0.0162	1.3	1.05, 1.66

Sig., Significant; eβ, Hazard Ratio (HR); CI, Confidence Interval.

Other Covariates*: clinical, hematology (Complete blood cell count (CBC)/Differential automated (Diff.), socio-demographic (supplemental Tables 1, 2) and socio-behavioral risk factors (supplemental Tables 3, 4) were used to control for possible confounding or interaction.

### Statistical analyses

According to the sample size, normality of data was checked by using ***Shapiro–Wilk-***test. Also, using independent ***t***-test to compare between continuous variables and using ***chi-square***-test to compare categorical data. Continuous variables were expressed as mean ± SD and categorical variables were expressed as percent (number).

Statistical analyses used descriptive and survival analyses (such as, Kaplan–Meier, Cox’s proportional hazard models for univariate, bivariate and multivariate). Kaplan–Meier for plotting trends of CD4 + T-lymphocyte decline and HIV progression associations by gender. Univariate, Bivariate and Multivariate Cox’s proportional hazard model were used to adjust for a number of covariates simultaneously to estimate the hazard ratio (HR) of CD4 + T-cell decline and HIV progression by gender and, taking into account the time between the initial CD4 + base value and the latest immunologic status (endpoint CD4 + T-lymphocyte < 500/µƖ). Data analysis is accomplished by utilizing the advanced version of SPSS.

### Informed consent

Informed consent was obtained from each participant.

## Results

Among eligible HIV-infected patients 85%, (40.0%, > 700/µƖ, 45.0% range 550–700/µƖ) with higher CD4 + T-lymphocyte counts and only 15%, range 500–549/µƖ) were identified for the study.

Table 2 shows females mean CD4 + base value is greater than males (741.9 vs.712.2, *P* = *0.421*). The mean of the length of time from initial HIV positive diagnosis to the first CD4 + T-lymphocyte count, is similar in both genders (7.45 vs. 7.20). However, females mean length of follow-up time for CD4 + decline is shorter than males (21.9 vs. 25.5), as they had a more rapid CD4 + T-cell decline. Mean CD4 + T-lymphocyte decline is approximately 1.5 times greater in females than males (234.5 vs. 158.6). Despite higher CD4 + base values and shorter follow-up times for females compared to males this difference was significant, (95% CI: − 127.93, − 23.93*, P* = ***0.004***).

In Table 3 results identified significantly that HIV-infected females progressed more rapidly than males (41.7% vs.24.6%, *P* < *0.02*).

Survival analysis results were laid out by specific aims and sub-hypotheses content. Before we began to analyze numeric results, we observed survival curves that displayed the trend of gender differences and CD4 + T-lymphocyte decline by Kaplan–Meier plots and Hazard Function Models.

This allowed for better examination of functions and comparison of gender role in CD4 + T-lymphocyte decline in a clear way, which leads us to assess rapid HIV progression more accurately in females compared to males. In fact, Kaplan–Meier plots identified females CD4 + T-lymphocyte decline is more rapid and leads to HIV progression compared to males. This indicates the longer the trend, the larger the differences of survival and vice versa (Figs. 1, 2).

Furthermore, Hazard model identified women's longevity is shorter than men (Fig. 3).

Table 4 shows the initial hematology-laboratory results. Initial WBC results identified no significant differences between females and males (95% CI: -0.02, 1.10, *P* = 0.059). Lymphocyte percent identified no significant results for females and males (95%CI: -2.74, 4.74, *P* = 0.599). However, platelets were significantly higher for females compared to males (95% CI: -51.32,-7.34, *P* = *0.001*), but fell into normal reference ranges (150–450 × 10^3^/µƖ) and these differences lead to a more rapid progression of HIV in females.

Moreover, numerous factors such as socio-demographic, socio-behavioral, clinical manifestations, biological, environmental, laboratory and frequency of infectious diseases presentation that may affect the natural history of HIV progression between or within genders as covariates simultaneously in univariate, bivariate and multivariate modeling were assessed by Cox’s proportional-hazard models. Consequently, models in Table 5 shows female gender increased the risk of CD4 + T-Lymphocyte decline than male.

In this study clinical observation suggests that HIV progression in females is more rapid compared to males. Univariate analysis identified that female gender increases the risk of CD4 + decline by 40% (HR = 1.4; 95%CI 0.96, 2.06). The relation between CD4 + T-Lymphocyte “*as a component of the WBC*” base value (***in hundreds***) and HIV progression reached significant (HR = 0.9; 95%CI 0.68, 0.88). On the other hand, the significance hazard ratio and confidence interval calculations for one-unit change for CD4 + base value (HR = 0.9; 95%CI 0.88, 0.99) are close to one.

In terms of bivariate analysis, we examined risk factors which may play a role in observed differences in HIV progression associated with gender. As anticipated, bivariate analysis was similar to univariate analysis, identified hazard ratio was not remarkably changed when gender was examined with the other single covariate separate models (HR = 1.4; 95%CI 0.97, 2.08).

Building the best model and getting similar answers for the variables of interest, related to the main hypotheses, determines the multivariate model that fits the data. Therefore, before applying multivariate analysis it is necessary to confirm the following clarifications. Utilizing the Wald-Forward-Stepwise analysis, there was no change in regression coefficients and no major change in relation to gender and CD4 + decline, by adding more variables to the model. Adding other variables and getting a similar answer for female gender and CD4 + decline, signifies the stability of the model and the best fit.

Consequently, the relationship between covariates and the outcome variable, as indicated in this model, are remarkably consistent with separate models of univariate and bivariate and quite reasonably stable condition for female gender and rapid CD4 + decline. At the same time, we test for interaction of gender and other covariates in the model, and “no interaction” was noted. However, few suspicious variables and discrepancy in HR estimated were not seen for univariate, bivariate, and interaction analyses. For real interaction we performed stratified analysis to detect the actual effect of these variables. However, there were no existing effects.

Hence, by considering all the variables, analysing them in various ways and steps to approve different routes and ending up with a similar answer for our main hypotheses, we are assured that multivariate Cox’s proportional-hazard models are the best fit in our analyses.

The result of multivariate modeling, after including all covariates assessments significantly identified that female gender increases the HR of HIV progression by two-fold (HR = 1.93; 95% CI:1.24, 2.99).

## Discussion

In this study, we hypothesized that CD4 + T-lymphocyte decline during follow-up time leads to HIV progression more rapidly in females than in males.

In this study, a significantly higher proportion of HIV-infected females (41.7%) were rapid progressor than males (24.6%) over a shorter follow-up period (*P* < 0.02).

We examined a variety of risk factors which may play a role in observed differences in HIV progression. Thus, after adjusting for different variables in the models in various ways, results did not alter the magnitude of female gender differences in rapid HIV progression. Therefore, a major finding of this study was that females had rapid HIV progression than males despite higher absolute CD4 + T-cell and shorter follow-up time due to more rapid CD4 + decline among females. In study subjects, the mean average CD4 + T-lymphocyte decline in crude analysis for females was approximately 1.5 times higher than males.

Based upon looking at all angles of the variables related to the main question, and having similar results from previous analyses, such as Kaplan–Meier graphical plot, univariate, bivariate, everything fits with our hypotheses. In univariate analysis and bivariate models female gender increased the risk of CD4 + T-cell decline by 40%.

After adjusting gender with all covariates and testing by multivariate Cox’s proportional hazard model, we obtain significantly stronger findings with a higher magnitude of 2-fold hazard ratio for females’ rapid CD4 + T-cell decline in shorter follow-up time and HIV progression compared with males over the 10-year period.

For further certainty, overall analyses of this study, from the repeated steps of analyses such as examining for possible potential confounding, interaction and effect modification of other extraneous variables in different models, results indicate the stability of the association of predictor (gender) and outcome variables (HIV progression) in our specific question. These findings are more concrete and consistent with previous studies^12,13,19,20,28^.

One study^29^ identified that women presented with higher absolute CD4 + T-lymphocyte counts than men, however, this difference was not significant. This discrepancy was related to components of their specific question.

Our finding is supported by many other studies and clinician experiences that enumerations of CD4 + T-lymphocytes remain the best immunological steady-state marker of HIV progression and prognosis^30–32^.

For more signifying, since the absolute CD4 + count is calculated based on WBC and lymphocyte percent values, the results of initial WBC and lymphocyte percentage were analyzed. In terms of the magnitude of CD4 + T-lymphocyte base value, change in one unit apart, results in the hazard ratio and confidence interval close to one. This indicates the issue of the size of the unit for CD4 + T-lymphocyte count, by changing one unit in the risk, is thus based upon one unit apart from the CD4 + T-lymphocyte base covariate. When we change the size of the CD4 + T-lymphocyte base value unit from one to 100 (because CD4 + T-lymphocyte baseline value counts are in the several hundred), the estimate of hazard ratio for CD4 + T-lymphocytes is far below one. Scientifically, this means that the CD4 + T-lymphocyte as a helper cell and immune protection declines more rapidly in females and causes a rapid HIV progression compared to males. Therefore, biological differences between genders have a clear influence on the natural history of HIV disease and the response to therapeutic interventions.

Furthermore, in this study the risk of CD4 + decline and HIV progression in females was approximately two-fold higher than in males, and recent results by other researchers^12,19,33^ parallel to this finding, considerations for changes in therapy protocols may need to be made to account for gender differences. Hence, findings of this study may have important clinical applications and epidemiologic significance.

## Limitations

HIV infection data should be interpreted with caution as these data may not be representative of all HIV-infected patients. The number of cases appears to be smaller than expected in the early phases of the study. Therefore, the unequal costs of this type of deficit may not be serious for study subjects due to satisfaction of results. Although the total sample size is not large and females are fewer than males, the obtained results are deemed reliable due to cohort study design and use of appropriate statistical methods to achieve enough power in this study. Increasing the sample size may have a minor impact on the covariate results but not a major impact on the nature of the predictor variable, generalizability, and conclusiveness of the analyses.

Errors from the diagnostic laboratory or clinic (mislabeling of blood specimens, etc.) and systematic errors (test kit, technical or procedural errors, equipment, or contamination of specimens) may lead to limitations and affect the magnitude of hazard ratio. However, due to directed supervision, quality assurance (QA) and quality control (QC) we assume to minimize these potential inaccuracies.

Delayed diagnosis may vary due to patients not seeking medical care and/or “no show” of clinic appointments has minor affects due to the precise measure of HIV progression. Barriers to access to care was postulated to be the reason for the observed gender differences^34^. Therefore, in this study due to thorough management and coordination of the WNY regional area, HIV/AIDS Referral Center at ECMC ensured that all patients received equal access, acceptability, availability, continuity of care, cost-effectiveness, and quality of care.

## Strengths

Study strengths demonstrate possible associations between those risk factors (real or related) and HIV progression by gender.

Minimizing potential biases by frequent QA-QC through all stages of data collection, analyses and cautiously interpreting data related to eligible subjects. Re-evaluation of collected data by the principle investigator in order to re-examine the outcome variable (CD4 + decline) in study subjects, from CD4 + baseline value to study endpoint (CD4 < 500/µƖ) by employing the absolute count mathematical computation formula used by the ECMC advanced level laboratory^35^. Each CD4 + lab test result was re-calculated by the principle investigator for verification of accuracy, validity, reliability of collected data.

Further strength is collected data from diverse geographically normal distributed centers that were providing health care with an abundance of information representative of the targeted WNY HIV-infected cohort population.

Another major benefit in obtaining valid CD4 + T-cell counts, on the study subjects, is that ECMC with advanced lab equipment (e.g. Flow Cytometry device, …) had the capacity to run all tests without having to send the specimens out for evaluation, that mitigate the risk of inter-laboratory measurement variations and altered results^35^.

## Conclusion

Although the biological mechanism remains unknown, findings suggest there are gender differences in CD4 + decline, with a higher risk of rapid HIV progression and shorter longevity in females.

## Supplementary information

Supplementary Information.
