# Supplementary material for: The rapid CD4 + T-lymphocyte decline and human immunodeficiency virus progression in females compared to males
Source: Sci Rep. 2020 Oct 8;10:16816. doi: 10.1038/s41598-020-73852-0 (PMC7544823; doi:10.1038/s41598-020-73852-0)
Supplement: Supplementary file 1 — Supplementary Information. [file 41598_2020_73852_MOESM1_ESM.pdf]

The rapid CD4+T-Lymphocyte decline and Human Immunodeficiency Virus progression in females compared to males

Nader Parsa[1], Pari Mahlagha Zaheri[2], Ross G Hewitt[3], Ali Karimi Akhormeh[1], Samira Taravatmanesh[2], Lisa Wallin[4]*

[1]Cardiovascular Research Center, Shiraz University of Medical Sciences, Shiraz, Iran.
[2]Shiraz University of Medical Sciences, Shiraz, Iran.
[3]University at Buffalo, State University of New York, Buffalo, New York, USA.
[4]Strong Memorial Hospital, University of Rochester, Rochester, New York, USA.

****Corresponding author***: Lisa Wallin, University of Rochester, Rochester, NY, USA.
Email: drnaderparsabe930@gmail.com. Cell phone. (585) 705-2072

Supplemental table-1 shows **socio-demographic characteristics** of HIV-infected males compared with females.

Supplemental Table-1: Socio-Demographic Characteristics for HIV-Infected Males (n=118) Compared with HIV-Infected Females (n=60)

Variable		Male %(n=118)	Female %(n=60)	Total %(n=178)	χ^2	*p-value*
Employment:	Unemployed	39.9 (47)	80.0 (48)	53.3 (95)	27.0	0.0001
	Employed	58.5 (69)	18.3 (11)	44.9 (80)		
	Disability	0.8 (1)	1.7 (1)	1.2 (2)		
	Unknown	0.8 (1)	0.0 (0)	0.6 (1)		
	Total:	100.0 (118)	100.0 (60)	100.0 (178)		
Insurance:	No insurance	18.6 (22)	18.3 (11)	18.5 (33)	12.8	0.047
	Private	28.9 (34)	10.0 (6)	22.5 (40)		
	Medicaid	46.6 (55)	66.7 (40)	53.5 (95)		
	Medicare	1.7 (2)	0.0 (0)	1.1 (2)		
	HMO	0.0 (0)	0.0 (0)	0.0 (0)		
	Self-pay	0.0 (0)	0.0 (0)	0.0 (0)		
	Medicaid & Medicare	3.4 (4)	3.3 (2)	3.2 (6)		
	Veteran's insurance	0.8 (1)	0.0 (0)	0.6 (1)		
	Medical satellite	0.0 (0)	1.7 (1)	0.6 (1)		
	Total:	100.0 (118)	100.0 (60)	100.0 (178)		
Race:	Black	36.4 (43)	38.3 (23)	37.1 (66)	3.4	0.335
	White	45.8 (54)	36.7 (22)	42.7 (76)		
	Hispanic	17.8 (21)	23.3 (14)	19.6 (35)		
	Other	0.0 (0)	1.7 (1)	0.6 (1)		
	Total:	100.0 (118)	100.0 (60)	100.0 (178)		
Marital Status	Single	71.1 (84)	48.3 (29)	63.5 (113)	21.8	0.001
	Married	10.2 (12)	18.3 (11)	12.9 (23)		
	Widowed	0.0 (0)	10.0 (6)	3.4 (6)		
	Divorced	10.2 (12)	10.0 (6)	10.1 (18)		
	Relationship with Significant Other	1.7 (2)	8.3 (5)	3.9 (7)		
	Separated	6.8 (8)	5.0 (3)	6.2 (11)		
	Total:	100.0 (118)	100.0 (60)	100.0 (178)		
Living Status:	Alone	33.9 (40)	20.0 (12)	29.2 (52)	15.1	0.035
	Nuclear family(husband, wife, unmarried children)	29.7 (35)	58.3 (35)	39.3 (70)		
	With Significant Other	22.1 (26)	11.7 (7)	18.6 (33)		
	With Other Relations	1.7 (2)	1.7 (1)	1.7 (3)		
	With Friend	5.1 (6)	1.7 (1)	3.9 (7)		
	Group Home /Domiciliary	4.2 (5)	3.3 (2)	3.9 (7)		
	Homeless	0.8 (1)	0.0 (0)	0.6 (1)		
	Unknown	2.5 (3)	3.3 (2)	2.8 (5)		
	Total:	100.0 (118)	100.0 (60)	100.0 (178)		

Supplemental Table-2 **Shows bivariate analysis of gender** with **sociodemographic risk** factors. Magnitude of association between gender mutual with sociodemographic and CD4+T-cell **decline remains unchanged** after adjusting for **employment insurance, race, marital status, living status, age and education**.

Supplemental Table-2: Bivariate Modelling of Gender with Socio-Demographic Risk Factors Their Impact on CD4 Decline and HIV Progression to AIDS in Western New York HIV-Infected Patients

Models		Statistical Analysis								
	Variable	*n*	*Events %(n)*	*Censored %(n)*	*ß (Beta)*	*SE(B)*	*[B/SE(B)]2*	Sig.	HR (*eß*)	*95%CI for eß*
Model 1	*Gender :*	178	67(119)	33 (59)						
	0.Males	118			0	1	Referent	0	1	Referent
	1.Femals	60			0.491	0.221	4.935	*0.02*	*1.6*	*1.05,2.52*
	Employment status:	178	67(119)	33 (59)						
	0.Unemploy	98			0	1	Referent	0	1	Referent
	1.Employ	80			0.307	0.210	2.129	0.14	1.4	0.90, 2.05
Model 2	*Gender :*	178	67(119)	33 (59)						
	0.Males	118			0	1	Referent	0	1	Referent
	1.Femals	60			0.339	0.201	2.824	*0.09*	*1.4*	*0.94,2.08*
	Insurance:	178	67(119)	33 (59)						
	0.Private	40			0	1	Referent	0	1	Referent
	1.Not Private	138			0.016	0.223	0.005	0.94	1.1	0.65, 1.57
Model 3	*Gender :*	178	67(119)	33 (59)						
	0.Males	118			0	1	Referent	0	1	Referent
	1.Femals	60			0.362	0.198	3.332	*0.06*	*1.4*	*0.97, 2.11*
	Race :	178	67(119)	33 (59)						
	0.White	76			0	1	Referent	0	1	Referent
	1.Non-white	102			-0.093	0.188	0.243	0.62	0.9	0.62, 1.31
Model 4	*Gender :*	178	67(119)	33 (59)						
	0.Males	118			0	1	Referent	0	1	Referent
	1.Femals	60			0.319	0.209	2.335	0.12	1.4	0.91, 2.07
	Marital Status:	178	67(119)	33 (59)						
	0.Single	113			0	1	Referent	0	1	Referent
	1.Married	65			0.061	0.201	0.093	0.75	1.1	0.71,1.57
Model 5	*Gender :*	178	67(119)	33 (59)						
	0.Males	118			0	1	Referent	0	1	Referent
	1.Femals	60			0.345	0.200	2.972	0.08	*1.4*	*0.95,2.09*
	Living Status:	178	67(119)	33 (59)						
	0.Alone	52			0	1	Referent	0	1	Referent
	1.Not alone	126			-0.010	0.206	0.002	0.96	0.9	0.66, 1.48
Model 6	*Gender :*	178	67(119)	33 (59)						
	0.Males	118			0	1	Referent	0	1	Referent
	1.Femals	60			0.380	0.196	3.735	*0.05*	*1.4*	*0.99,2.15*
	Age	178	67(119)	33 (59)	0.014	0.012	1.387	0.23	1.0	0.99,1.04
Model 7	*Gender :*	178	67(119)	33 (59)						
	0.Males	118			0	1	Referent	0	1	Referent
	1.Femals	60			0.343	0.195	3.086	*0.07*	*1.4*	*0.96, 2.06*
	Education:	178	67(119)	33 (59)						
	0. ≤High School	136			0	1	Referent	0	1	Referent
	1. ≥College	42			-0.001	0.220	0.000	0.99	0.9	0.64, 1.53

Sig. =Significant, e ß= Hazard Ratio (HR))

It is known that several adverse **socio-behavioral** characteristics play a role in the acquisition of HIV infection, but their contribution to HIV progression is unclear. Therefore, we hypothesize that there is a gender difference in the distribution of socio-behavioral risk factors between females and males. However, we evaluated those possible risk factors in our cohort study (see *Supplemental Table-3).*

Supplemental table-3: General Socio-Behavioural, Sexual Orientation, Sexual Contact with AIDS-Risk Member Characteristics , and Protected Sex During the Follow-up Time for HIV-Infected Males (n=118) Compared with HIV-Infected Females (n=60)

Variable		Male %(n=118)	Female %(n=60)	Total %(n=178)	χ^2	*p-Value*
Sexual Orientation, AIDS-Risk Member Sexual Partner	Homosexual	36.4 (43)	0.0 (0)	24.2 (43)	75.45	0.0001
	Heterosexual	19.5 (23)	21.8(13)	20.2(36)		
	Bisexual	11.9 (14)	0.0 (0)	7.9 (14)		
	Intravenous Drug Use (IVDU)Partner	6.8(8)	30.0(18)	14.6(26)		
	Homosexual& IVDU	6.8 (8)	0.0 (0)	4.5 (8)		
	Heterosexual& IVDU	14.4(17)	41.6 (25)	23.6(42)		
	Bisexual & IVDU	4.2 (5)	6.6 (4)	5.0 (9)		
	Total:	100.0 (118)	100.0 (60)	100.0 (178)		
Protected Sex:	No	75.4 (89)	56.7(34)	69.1(123)	7.6	0.023
	Yes	23.8(28)	43.3(26)	30.3(54)		
	Unknown	0.8 (1)	0.0(0)	0.6(1)		
	Total:	100.0 (118)	100.0 (60)	100.0 (178)		
Alcohol Intake:	Never	15.2 (18)	30.8 (18)	20.3 (36)	16.5	0.011
	Light (<1 drink/day)	30.5 (36)	21.7 (13)	27.5 (49)		
	Moderate(1-3 drinks/day)	11.9 (14)	6.7 (4)	10.1 (18)		
	Heavy (> 4 drinks/day)	39.0 (46)	35.0 (21)	37.6 (67)		
	Unknown	3.4 (4)	6.6 (4)	4.5 (8)		
	Total:	100.0 (118)	100.0 (60)	100.0 (178)		
Amphetamines (Speed):	No/Never	92.4 (109)	95.0 (57)	93.3 (166)	6.5	0.039
	Yes	7.6 (9)	1.7 (1)	5.6 (10)		
	Unknown	0.0 (0)	3.3 (2)	1.1 (2)		
	Total:	100.0 (118)	100.0 (60)	100.0 (178)		
Lysergic Acid Diethylamide (*LSD*):	No/Never	94.1 (111)	93.4 (56)	93.8 (167)	4.5	0.107
	Yes	5.9 (7)	3.3 (2)	5.1 (9)		
	Unknown	0.0 (0)	3.3 (2)	1.1 (2)		
	Total:	00.0 (118)	00.0 (60)	100.0 (178)		
Intravenous Drug Use (*IVDU*):	No/Never	64.4 (76)	55.0 (33)	61.2 (109)	22.3	0.0001
	Past*	16.9 (20)	15.0 (9)	16.3 (29)		
	Current*	3.4 (4)	20.0 (12)	9.0 (16)		
	Past &Current*	15.3 (18)	5.0 (3)	11.8 (21)		
	Unknown	0.0 (0)	5.0 (3)	1.7 (3)		

Supplemental table-4 display **Bbivariate modelling** of gender mutual with sociobehavioral risk factors. No remarkable risk effect is observed for gender and different types of sexual orientation/ partnership, protected sex, amphetamine use, LSD, and IVDU.

Supplemental table-4: Bivariate Modelling of Gender with Socio-Behavioural (sexual orientation and sexual partners, protected sex, alcohol intake, illicit drug use, IVDU) Risk Factors, Their Impact on CD4 Decline and HIV Progression to AIDS in Western New York HIV-Infected Patients

Models		Statistical Analysis								
	Variable	*n*	*Events %(n)*	*Censored %(n)*	*ß (Beta)*	*SE(B)*	*[B/SE(B)]2*	Sig.	HR (*eß*)	*95%CI for eß*
Model 8	*Gender :* 0.Males 1.Females	178	67(119)	33 (59)						
		118			0	1	Referent	0	1	Referent
		60			0.309	0.229	1.828	0.18	1.4	0.86, 2.14
	Sexual Partner : 0.IVDU Partner 1.Homosexual 2.Heterosexual 3. Bisexual	178	67(119)	33 (59)						
		26			0	1	Referent	0	1	Referent
		51			0.147	0.337	0.190	0.66	1.1	0.59, 2.24
		78			0.298	0.298	1.063	0.30	1.3	0.76, 2.37
		23			-0.213	0.372	0.328	0.56	0.8	0.39, 1.67
Model 9	*Gender :* 0.Males 1.Females	178	67(119)	33 (59)						
		118			0	1	Referent	0	1	Referent
		60			0.332	0.198	2.800	0.09	1.4	0.94, 2.05
	Protected Sex: 0.No 1.Yes	178	67(119)	33 (59)						
		124			0	1	Referent	0	1	Referent
		54			0.058	0.210	0.076	0.78	1.1	0.70,1.59
Model 11	*Gender :* 0.Males 1.Females	178	67(119)	33 (59)						
		118			0	1	Referent	0	1	Referent
		60			0.398	0.202	3.894	0.04	1.5	1.00,2.21
	Alcohol Intake: 0.No 1.Yes	178	67(119)	33 (59)						
		36			0	1	Referent	0	1	Referent
		142			0.222	0.228	0.947	0.33	1.2	0.79,1.95
Model 12	*Gender :* 0.Males 1.Females	178	67(119)	33 (59)						
		118			0	1	Referent	0	1	Referent
		60			0.354	0.194	3.314	0.06	1.4	0.97, 2.08
	Amphetamine: 0.No 1.Yes	178	67(119)	33 (59)						
		168			0	1	Referent	0	1	Referent
		10			_0.407	0.391	1.085	0.29	0.7	0.30, 1.43
Model 13	*Gender :* 0.Males 1.Females	178	67(119)	33 (59)						
		118			0	1	Referent	0	1	Referent
		60			0.367	0.194	3.545	*0.05*	*1.4*	*0.98,2.11*
	Lysergic Acid Diethylamide (LSD): 0.No 1.Yes	178	67(119)	33 (59)						
		169			0	1	Referent	0	1	Referent
		9			-0.690	0.421	2.687	0.10	0.5	0.21, 1.14
Model 14	*Gender :* 0.Males 1.Female	178	67(119)	33 (59)						
		118			0	1	Referent	0	1	Referent
		60			0.361	0.195	3.421	*0.06*	*1.4*	*0.97,2.10*
	IVDU: 0.No 1.Yes	178	67(119)	33 (59)						
		112			0	1	Referent	0	1	Referent
		66			0.175	0.194	0.810	0.36	0.8	0.57, 1.22

Sig. =Significant, e ß= Hazard Ratio (HR)
